# ORCA-SPOT: An Automatic Killer Whale Sound Detection Toolkit Using Deep Learning

**DOI:** 10.1038/s41598-019-47335-w

**Published:** 2019-07-29

**Authors:** Christian Bergler, Hendrik Schröter, Rachael Xi Cheng, Volker Barth, Michael Weber, Elmar Nöth, Heribert Hofer, Andreas Maier

**Affiliations:** 1 Friedrich-Alexander-University Erlangen-Nuremberg, Department of Computer Science, Pattern Recognition Lab, Martensstr. 3, 91058 Erlangen, Germany; 2 Department of Ecological Dynamics, Leibniz Institute for Zoo and Wildlife Research (IZW) in the Forschungsverbund Berlin e.V., Alfred-Kowalke-Straße 17, 10315 Berlin, Germany; 3 Anthro-Media, Nansenstr. 19, 12047 Berlin, Germany; 4 Department of Biology, Chemistry, Pharmacy, Freie Universität Berlin, Takustrasse 3, 14195 Berlin, Germany; 5 Department of Veterinary Medicine, Freie Universität Berlin, Oertzenweg 19b, 14195 Berlin, Germany

**Keywords:** Animal behaviour, Behavioural ecology

## Abstract

Large bioacoustic archives of wild animals are an important source to identify reappearing communication patterns, which can then be related to recurring behavioral patterns to advance the current understanding of intra-specific communication of non-human animals. A main challenge remains that most large-scale bioacoustic archives contain only a small percentage of animal vocalizations and a large amount of environmental noise, which makes it extremely difficult to manually retrieve sufficient vocalizations for further analysis – particularly important for species with advanced social systems and complex vocalizations. In this study deep neural networks were trained on 11,509 killer whale (*Orcinus orca*) signals and 34,848 noise segments. The resulting toolkit ORCA-SPOT was tested on a large-scale bioacoustic repository – the Orchive – comprising roughly 19,000 hours of killer whale underwater recordings. An automated segmentation of the entire Orchive recordings (about 2.2 years) took approximately 8 days. It achieved a time-based precision or positive-predictive-value (PPV) of 93.2% and an area-under-the-curve (AUC) of 0.9523. This approach enables an automated annotation procedure of large bioacoustics databases to extract killer whale sounds, which are essential for subsequent identification of significant communication patterns. The code will be publicly available in October 2019 to support the application of deep learning to bioaoucstic research. ORCA-SPOT can be adapted to other animal species.

## Introduction

There has been a long-standing interest to understand the meaning and function of animal vocalizations as well as the structures which determine how animals communicate^1^. Studies on mixed-species groups have advanced the knowledge of how non-human primates decipher the meaning of alarm calls of other species^2,3^. Recent research indicates that bird calls or songs display interesting phonological, syntactic, and semantic properties^4–8^. In cetacean communication, whale songs are a sophisticated communication system^9^, as in humpback whales *(Megaptera novaeangliae)* whose songs were found to be only sung by males and mostly during the winter breeding season^10^. These are believed to attract prospective female mates and/or establish dominance within male groups^11,12^. Moreover, studies on captive and temporarily captured wild bottlenose dolphins *(Tursiops truncatus)* have shown that individually distinct, stereotyped signature whistles are used by individuals when they are isolated from the group^13–15^, in order to maintain group cohesion^16^.

Many different animal species have a strong ability to communicate. In this study, the killer whale was used as a prototype in order to confirm the importance and general feasibility of using machine-based deep learning methods to study animal communication.

**Figure 1:**
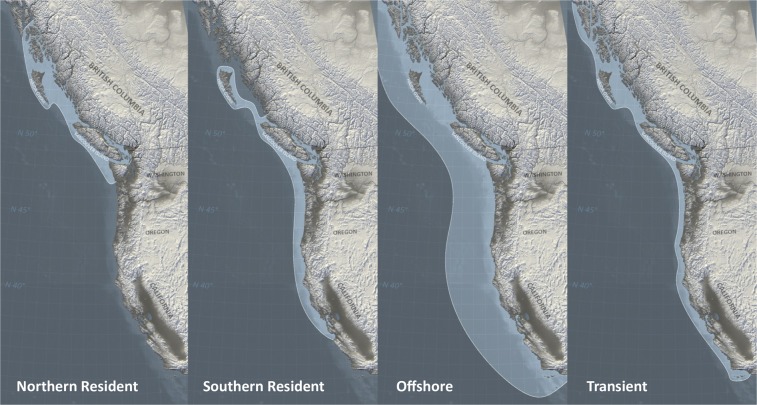
Geographic ranges (light shading) of killer whale populations in northeastern Pacific (British Columbia, Canada) (Illustration recreated after Wiles^31^).

**Figure 2:**
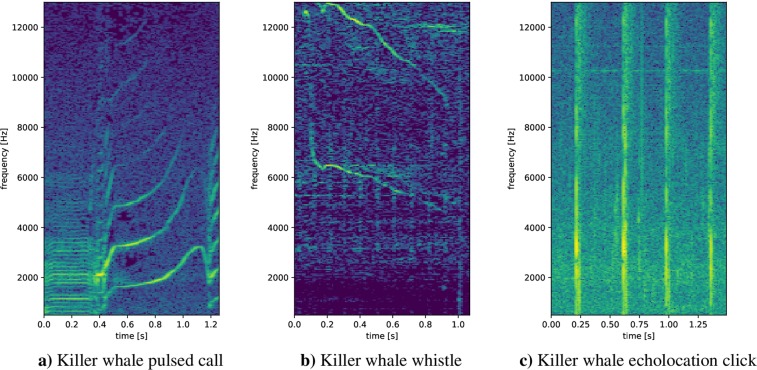
Spectrograms of three characteristic killer whale sounds (sampling rate = 44.1 kHz, FFT-size = 4,096 samples (≈100 ms), hop-size = 441 samples (≈10 ms)).

Killer whales (*Orcinus orca*) are the largest members of the dolphin family and are one of several species with relatively well-studied and complex vocal cultures^17^. Recent studies on killer whale and bottlenose dolphin brains reveal striking and presumably adaptive features to the aquatic environment^18–21^. They are believed to play an important role in their communicative abilities and complex information processing^22^. Extensive research on killer whale acoustic behavior has taken place in the Northeast Pacific where resident fish-eating, transient mammal-eating and offshore killer whales can be found, the three ecotypes of killer whales in this region. They differ greatly in prey preferences, vocal activity, behavior, morphology and genetics^23–27^. Figure 1 shows the population distribution and geographic ranges of killer whales in the Northeast Pacific. Resident killer whales live in stable matrilineal units that join together to socialize on a regular basis, forming subpods and pods^28,29^. Different pods produce distinct vocal repertoires, consisting of a mixture of unique and shared (between matrilines) discrete call types, which are referred to as dialects. Ford^30^ and Wiles^31^ suggested that individuals from the same matriline and originating from a common ancestor most likely share similar acoustic vocal behaviors. Pods that have one or more discrete calls in common are classified as one acoustic clan^32^. The diverse vocal repertoire of killer whales comprises clicks, whistles, and pulsed calls^33^. Like other odontocetes, killer whales produce echolocation clicks, used for navigation and localization, which are short pulses of variable duration (between 0.1 and 25 ms) and a click-repetition-rate from a few pulses to over 300 per second^33^ (Fig. 2a). Whistles are narrow band tones with no or few harmonic components at frequencies typically between 1.5 and 18 kHz and durations from 50 ms up to 12 s^33^ (Fig. 2b). As recently shown, whistles extend into the ultrasonic range with observed fundamental frequencies ranging up to 75 kHz in three Northeast Atlantic populations but not in the Northeast Pacific^34^. Whistles are most commonly used during close-range social interactions. There are variable and stereotyped whistles^35–37^. Pulsed calls, the most common and intensively studied vocalization of killer whales, typically show sudden and patterned shifts in frequency, based on the pulse repetition rate, which is usually between 250 and 2000 Hz^33^ (Fig. 2c). Pulsed calls are classified into discrete, variable, and aberrant calls^33^. Some highly stereotyped whistles and pulsed calls are believed to be culturally transmitted through vocal learning^36,38–41^. Mammal-hunting killer whales in the Northeast Pacific produce echolocation clicks, pulsed calls and whistles at significantly lower rates than fish-eating killer whales^36,42,43^ because of differences in the hearing sensitivity of their respective prey species^44^. The acoustic repertoire in terms of discrete calls of Northeast Pacific killer whales is made up of calls with and without a separately modulated high-frequency component^45^. The use of discrete calls, with and without an overlapping high-frequency component, was also observed in southeast Kamchatka killer whales^46^. In the Norwegian killer whale population, pod-specific dialects were reported^47^, and a number of call types used in different contexts were documented^47,48^, though much less is known about their vocalizations and social systems^49^.

With the decrease of hardware costs, stationary hydrophones are increasingly deployed in the marine environment to record animal vocalizations amidst ocean noise over an extended period of time. Bioacoustic data collected in this way is an important and practical source to study vocally active marine species^50–53^ and can make an important contribution to ecosystem monitoring^54^. One of the datasets that the current study uses is the Orchive^55,56^, containing killer whale vocalizations recorded over a period of 23 years and adding up to approximately 19,000 hours. Big acoustic datasets contain a wealth of vocalizations. However, in many cases the data density in terms of interesting signals is not very high. Most of the large bioacoustic databases have continuously been collected over several years, with tens of thousands of hours usually containing only a small percentage of animal vocalizations and a large amount of environmental noise, which makes it extremely difficult to manually retrieve sufficient vocalizations for a detailed call analysis^56,57^. For example, so far only ≈1.6% of the Orchive was partially annotated by several trained researchers. This is not only time consuming and labor intensive but also error-prone and often results in a limited sample size, being too small for a statistical comparison of difference^58^, and thus for the recognition of significant patterns. Both, the strong underrepresentation of valuable signals, and the enormous variation in the characteristics of acoustic noise are big challenges. The motivation behind our work is to enable a robust and machine-driven segmentation, in order to efficiently handle large data corpora and separate all interesting signal types from noise.

Before conducting a detailed call analysis, one needs to first isolate and extract the interesting bioacoustic signals. In the past decade, various researchers have used traditional signal processing and speech recognition techniques, such as dynamic time warping^59–61^, hidden Markov and Gaussian mixture models^62–65^, as well as spectrogram correlation^66,67^ to develop algorithms in order to detect dolphin, bowhead whale, elephant, bird, and killer whale vocalizations. Others have adopted techniques like discriminant function analysis^68,69^, random forest classifiers^70,71^, decision tree classification systems^72^, template-based automatic recognition^73^, artificial neural networks^74–77^, and support vector machines^56,78^ in conjunction with (handcrafted) temporal and/or spectral features (e.g. mel-frequency cepstrum coefficients) for bat, primate, bird, and killer whale sound detection/classification. Many of the aforementioned research works^59–67,69,72,74,75,77,78^ used much smaller datasets, both for training and evaluation. In addition, for many of those traditional machine-learning techniques, a set of acoustic (handcrafted) features or parameters needed to be manually chosen and adjusted for the comparison of similar bioacoustic signals. However, features derived from small data corpora usually do not reflect the entire spread of signal varieties and characteristics. Moreover, traditional machine-learning algorithms often perform worse than modern deep learning approaches, especially if the dataset contains a comprehensive amount of (labeled) data^79^. Due to insufficient feature qualities, small training/validation data, and the traditional machine-learning algorithms themselves, model robustness and the ability to generalize suffer greatly while analyzing large, noise-heavy, and real-world (unseen) data corpora containing a variety of distinct signal characteristics. Furthermore, traditional machine-learning and feature engineering algorithms have problems in efficiently processing and modelling the complexity and non-linearity of large datasets^80^. Outside the bioacoustic field, deep neural network (DNN) methods have progressed tremendously because of the accessibility to large training data and increasing computational power by the use of graphics processing units (GPUs)^81^. DNNs have not only performed well in computer vision but also outperformed traditional methods in speech recognition as evaluated in several benchmark studies^82–85^. Such recent successes of DNNs inspired the bioacoustic community to apply state-of-the-art methods on animal sound detection and classification. Grill^86^ adopted feedforward convolutional neural networks (CNNs) trained on mel-scaled log-magnitude spectrograms in a bird audio detection challenge. Other researchers also implemented various types of deep neural network architecture for bird sound detection challenges^79^ and for the detection of koala activities^87^. Google AI Perception recently has successfully trained a convolutional neural network (CNN) to detect humpback whale calls in over 15 years of underwater recordings captured at several locations in the Pacific^57^.

This study utilizes a large amount of labeled data and state-of-the-art deep learning techniques (CNN) effectively trained to tackle one main challenge in animal communication research: develop an automatic, robust, and reliable segmentation of useful and interesting animal signals from large bioacoustic datasets. None of the above mentioned previous studies focused on such an extensive evaluation in real-world-like environments, verifying model robustness and overall success in generalization under different test cases and providing several model metrics and error margins in order to prepare and derive a network model that will be able to support researchers in future fieldwork.

The results from this study provide a solid cornerstone for further investigations with respect to killer whale communication or any other communicative animal species. Robust segmentation results enable, in a next step, the generation of machine-identified call types, finding possible sub-units, and detecting reoccurring communication patterns (semantic and syntactic structures). During our fieldwork, conducted in British Columbia (Vancouver Island) in 2017/2018, video footage on killer whale behaviour of about 89 hours was collected. The video material, together with the observed behavioral patterns, can be used to correlate them with the derived semantic and syntactic communication patterns. This is a necessary step ahead towards deriving language patterns (language model) and further understanding the animals.

The well-documented steps and the source code^88^ will be made freely available to the bioacoustic community in October 2019. Other researchers can improve/modify the algorithms/software in order to use it for their own research questions, which in turn will implicitly advance bioacoustics research. Moreover, all segmented and extracted audio data of the entire Orchive will be handed over to the OrcaLab^55^ and Steven Ness^56^.

## Data Material

The following section describes all datasets used for network training, validation and testing. Table 1 gives a brief summary of all used datasets and provides an overview on the amount of data and sample distribution of each partition. Each data corpus consists of already extracted and labeled killer whale and noise audio files of various length. In order to use the illustrated labeled data material as network input, several data preprocessing and augmentation steps were processed as described in detail in the methods section. Each audio sample was transformed into a 2-D, decibel-scaled, and randomly augmented power spectrogram, corresponding to the final network input. The network converts each input sample into a 1 × 2 matrix reflecting the probability distribution of the binary classification problem – killer whale versus noise (any non-killer-whale sound).

**Table 1:** Overview datasets and data distribution.

split		training				validation				test			
		samples				samples				samples			
dataset		killer whale	noise	sum	%	killer whale	noise	sum	%	killer whale	noise	sum	%
OAC^b^	**11,504**	6,829	1,213	8,042	69.9	1,425	286	1,711	14,9	1,443	308	1,751	15.2
AEOTD^a^	**17,995**	1,289	13,135	14,424	80.2	276	1,511	1,787	9.9	102	1,682	1,784	9.9
DLFD	**31,928**	3,391	20,500	23,891	74.8	1,241	2,884	4,125	12.9	1,108	2,804	3,912	12.3
SUM	**61,427**	11,509	34,848	46,357	75.5	2,942	4,681	7,623	12.4	2,653	4,794	7,447	12.1

^a^Dataset available upon request^55,56^.

^b^Orchive tapes available upon request^55,56^.

### Orchive annotation catalog (OAC)

**Figure 3:**
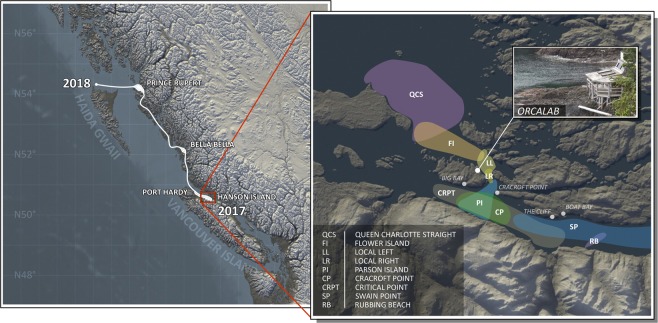
(**a**) (left) Expedition route and data collection range of DeepAL project 2017/2018 (**b**) (right) A network of hydrophones and the acoustic range of the OrcaLab^55^ (Illustration b) recreated after OrcaLab^55^ and Ness^56^).

The Orchive^55,56^ was created by Steven Ness^56^ and the OrcaLab^55^, including 23,511 tapes each with ≈45-minute of underwater recordings (channels: stereo, sampling rate: 44.1 kHz) captured over 23 years in Northern British Columbia (Canada) and summing up to 18,937.5 h. The acoustic range of the hydrophones covers the killer whales’ main summer habitats in Johnstone Strait (British Columbia, Canada) by using 6 radio-transmitting, various custom-made stationary hydrophones having an overall frequency response of 10 Hz–15 kHz^89^. A two-channel audio cassette recorder (Sony Professional, Walkman WM-D6C or Sony TCD-D3) was used to record the mixed radio receiver output by tuning to frequencies of the remote transmitters^89^. The entire hydrophone network was continuously monitored throughout day and night during the months when Northern Resident killer whales generally visit this area (July – Oct./Nov.) and was manually started when killer whales were present. Based on the Orchive, the OrcaLab^55^, Steven Ness^56^, and several recruited researchers extracted 15,480 human-labeled audio files (Orchive Annotation Catalog (OAC)) through visual (spectrogram) and aural (audio) comparison, resulting in a total annotation time of about 12.3 h. The Orchive tape data, as well as the OAC corpus, is available upon request^55,56^. A more detailed overview about the recording territory of OrcaLab^55^ is shown in Fig. 3b. The annotations are distributed over 395 partially-annotated tapes of 12 years, comprising about 317.7 h (≈1.68% of the Orchive). The killer whale annotations contain various levels of details, from labels of only echolocation clicks, whistles, and calls to further knowledge about call type, pod, matriline, or individuals. The original OAC corpus contains 12,700 killer whale sounds and 2,780 noise clips. Of about 12,700 labeled killer whale signals only ≈230 are labeled as echolocation clicks, ≈40 as whistles, and ≈3,200 as pulsed calls. The remaining ≈9,230 killer whale annotations are labeled very inconsistently and without further differentiation (e.g.“orca”, “call”) and therefore do not provide reliable information about the respective killer whale sound type. The annotated noise files were split into human narrations and other noise files (e.g. boat noise, water noise, etc.). Human voices are similar to pulsed calls considering the overlaying harmonic structures. For a robust segmentation of killer whale sounds human narrations were excluded. Furthermore, files that are corrupted, mislabeled or have bad qualities were excluded. Summing up, 11,504 labels (9,697 (84.3%) killer whale, 1,807 (15.7%) noise) of the OAC corpus (Table 1) were used and split into 8,042 samples (69.9%) for training, 1,711 (14.9%) for validation and 1,751 (15.2%) for testing. Audio signals from each single tape were only stored in either train, validation or test set.

### Automatic extracted orchive tape data (AEOTD)

OAC has an unbalanced killer whale/noise distribution. As a solution, 3-second audio segments were randomly extracted from different Orchive tapes, machine-labeled by an early version of ORCA-SPOT, and if applicable manually corrected. The evaluation was done by listening to the machine-segmented underwater signals as well as verifying the respective spectrograms in parallel. In total this semi-automatically generated dataset (AEOTD) contains 17,995 3-second audio clips. AEOTD consisted of 1,667 (9.3%) killer whale and 16,328 (90.7%) noise files. During validation, very weak (silent) parts (no underwater noise or any noticeable signal) of the tapes as well as special noises (e.g. microphone noises, boat noises, etc.), which are not part of the OAC corpus, were increasingly detected as killer whales, contributing to a growing false-positive-rate. Therefore, very weak (silent) audio samples were added to the training set only. As for OAC the 17,995 samples were split into 14,424 (80.2%) training, 1,787 (9.9%) validation and 1,784 (9.9%) test clips (Table 1). Similarly, annotations from each single tape were only stored in one of the three sets.

### DeepAL fieldwork data 2017/2018 (DLFD)

The DeepAL fieldwork data 2017/2018 (DLFD)^90^ were collected via a 15-m research trimaran in 2017/2018 in Northern British Columbia by an interdisciplinary team consisting of marine biologists, computer scientists and psychologists, adhering to the requirements by the Department of Fisheries and Oceans in Canada. Figure 3a visualizes the area which was covered during the fieldwork expedition in 2017/2018. A custom-made high sensitivity and low noise towed-array was deployed, with a flat frequency response of within ±2.5 dB between 10 Hz and 80 kHz. Underwater sounds were digitized with a sound acquisition device (MOTU 24AI) sampling at 96 kHz, recorded by PAMGuard^91^ and stored on hard drives as multichannel wav-files (5 total channels, 4 hydrophones in 2017 plus 1 additional channel for human researchers; 24 total channels, 8 channels towed array, 16 channels hull-mounted hydrophones in 2018). The 2017/2018 total amount of collected audio data comprises ≈157.0 hours. Annotations on killer whale vocalizations were made by marine biologists through visual and aural comparison using Raven Pro 1.5^92^ and John Ford’s^30^ call type catalog. In total the labeled 2017/2018 DeepAL fieldwork data (DLFD)^90^ includes 31,928 audio clips. The DLFD datset includes 5,740 (18.0%) killer whale and 26,188 (82.0%) noise labels. The total amount of 31,928 audio files was split into 23,891 (74.8%) train, 4,125 (12.9%) validation, and 3,912 (12.3%) test samples (Table 1), whereas samples of different channels of a single tape were only stored in one set.

## Results

**Figure 4:**
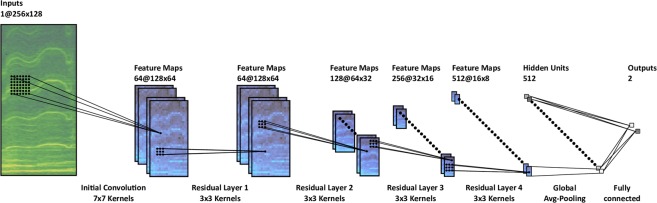
ORCA-SPOT network architecture.

The results are divided into three sections. The first section investigates the best ORCA-SPOT network architecture (Fig. 4). Once the architecture was chosen, ORCA-SPOT was trained, validated and tested on the dataset listed in Table 1. Validation accuracy was the basis for selecting the best model. First, two model versions of ORCA-SPOT (OS1, OS2) were verified on the test set. OS1 and OS2 utilized identical network architectures and network hyperparameters. Both models only differed in the number of noise samples included in the training set and the normalization technique used within the data preprocessing pipeline (dB-normalization versus mean/standard deviation (stdv) normalization). Due to identical network setups and an inconsistent training data corpus, the main intention of such a model comparison was not to directly compare two different networks, but rather illustrating the proportion of changing network independent parameters in order to further improve the overall model generalization and (unseen) noise robustness. In a second step we ran OS1 and OS2 on 238 randomly chosen ≈45-minute Orchive tapes (≈191.5 h audio), calculating the precision. Additionally OS1 and OS2 were evaluated on 9 fully-annotated, ≈45-minute Orchive tapes, which were chosen based on the number of killer whale activities. The AUC metric was used to determine the accuracy of classification.

### Network architecture

ORCA-SPOT was developed on the basis of the well-established ResNet architecture^93^. Two aspects were reviewed in greater detail: (1) traditional ResNet architectures with respect to their depth and (2) removal/preservation of the max-pooling layer in the first residual layer. The behavior of deeper ResNet architectures in combination with the impact of the max-pooling layer (3 × 3 – kernel, stride 2) in the first residual layer were examined in a first experiment. ResNet18, ResNet34, ResNet50, and ResNet101 were used as common ResNet variants. All these traditional and well-established network architectures are described in detail in the work of He *et al*.^93^. Each model was trained, developed and tested on the dataset illustrated in Table 1 in order to handle the binary classification problem between killer whale and noise. The test set accuracy, using a threshold of 0.5 (killer whale/noise), was chosen as a criterion for selecting the best architecture. In three evaluation runs under equal conditions (identical network hyperparameters, equal training/validation/test set, and same evaluation threshold) the max-pooling option was investigated together with various ResNet architectures. Random kernel-weight initializations and integrated on-the-fly augmentation techniques led to slight deviations with respect to the test accuracy of each run. For each option and respective ResNet model, the maximum, mean, and standard deviation of all three runs was calculated. Table 2 shows that deeper ResNet models do not necessarily provide significant improvements on test set accuracy. This phenomenon can be observed in cases of removing or keeping max-pooling. Models without max-pooling in the first residual layer displayed an improvement of ≈1% on average. Furthermore, the marginal enhancements of the averaged test set accuracy during the application of deeper ResNet architectures resulted in much longer training times on an Nvidia GTX 1080 (ResNet18 = ≈4 h, ResNet34 = ≈6 h, ResNet50 = ≈8 h, ResNet101 = ≈10 h). Apart from the training time, the inference time of deeper networks was also significantly longer. ResNet18 processed an Orchive tape of ≈45-minutes length within about 2 minutes. ResNet34 took about 3.5 minutes and ResNet50 lasted about 5 minutes, resulting in a real-time factor of 1/13 and 1/9 compared to ResNet18 with 1/25. The entire Orchive (≈19,000 hours) together with four prediction processes (Nvidia GTX 1050) running in parallel resulted in a computation time of eight days for ResNet18, 14 days for ResNet34, and 20 days for ResNet50. Compared to ResNet18, none of the deeper ResNet architectures led to a significant improvement in terms of mean test set accuracy. ResNet18 performed on average only ≈0.5 percent worse than the best architecture (ResNet50) but was more than twice as fast relating to training and inference times. For all other ResNet architectures, the differences in accuracy were even smaller. As the final network architecture, ResNet18 without max-pooling in the first residual layer was chosen, in order to maximize the trade-off between accuracy and training/inference times. In particular, the second aspect is very important in terms of using the software on the vessel in the field. Due to limited hardware and the requirement to parse the incoming audio data in quasi real-time (killer whale versus noise), a good network performance is of essential importance. ResNet18 performs well, even on a mid-range GPU.

**Table 2:** Model accuracies for common ResNet architectures by comparing architectures with and without max pooling (3 × 3 kernel, stride 2) in the first residual layer.

Model	ORCA-SPOT-MAX-POOL						ORCA-SPOT-NO-MAX-POOL					
	Accuracy %			Statistics %			Accuracy %			Statistics %		
Arch	run1	run2	run3	max	mean	stdv	run1	run2	run3	max	mean	stdv
ResNet18	95.39	93.99	92.84	95.39	94.08	1.28	95.88	96.15	94.40	96.15	95.48	0.94
ResNet34	93.65	95.72	95.20	95.72	94.86	1.08	96.13	95.65	95.12	96.13	95.64	0.51
ResNet50	92.39	95.76	94.88	95.76	94.35	1.75	96.37	95.90	95.61	96.37	95.96	0.38
ResNet101	94.39	95.33	95.01	95.33	94.91	0.47	95.81	94.10	96.24	96.24	95.39	1.13

### ORCA-SPOT – training/validation/test set metrics

**Figure 5:**
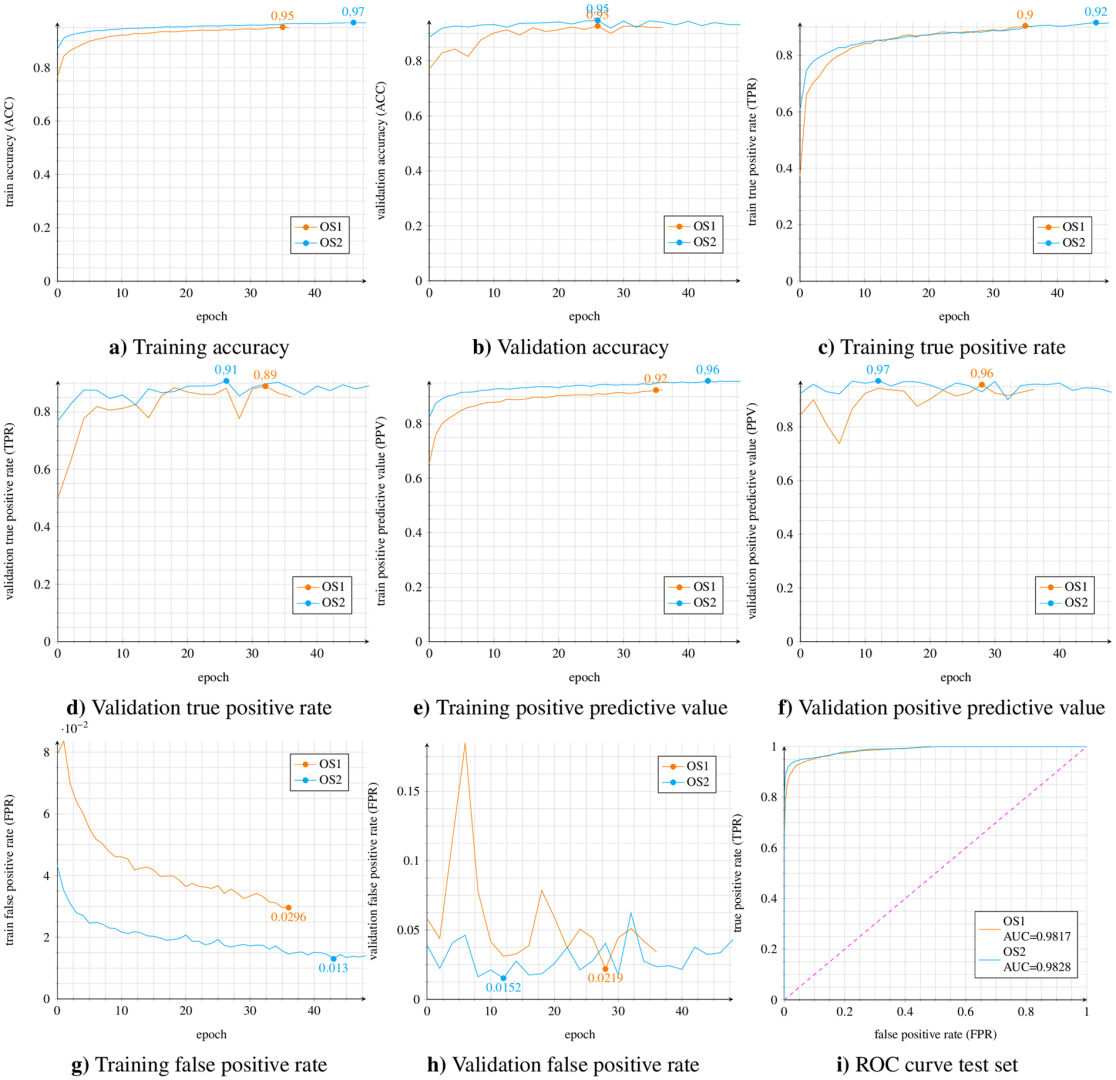
ORCA-SPOT training, validation and test set metrics (Table 1).

This section describes in detail the training, validation, and testing process of two different models, named ORCA-SPOT-1 (OS1) and ORCA-SPOT-2 (OS2). Both models depend on the same modified ResNet18 architecture and used identical network hyperparameters. During the entire training and validation phase the following metrics were evaluated: classification accuracy (ACC), true-positive-rate (TPR, recall with respect to “killer whale”), false-positive-rate (FPR), and positive-predictive-value (PPV, precision with respect to “killer whale”). The AUC was used to describe the test set results. All metrics, calculated after every epoch, are visualized in Fig. 5. OS2 implements a dB-normalization (min = −100 dB, ref = +20 dB) between 0 and 1, whereas OS1 includes a mean/stdv – normalization approach. Especially tapes without any noticeable underwater/killer whale sound activities led to extreme values regarding the mean/stdv – normalization due to a standard deviation close to zero causing higher false positive rates. To counteract this problem of very weak (silent) signals a dB-normalization was performed within a fixed range (0–1). OS2 was trained on the training set displayed in Table 1. The training set of OS2 differs from the training set of OS1 by containing 6,109 additional noise samples in the AEOTD corpus. The main motivation was to further improve the generalization and noise robustness of the model by adding more additional unseen noise samples. Those noise samples were previously represented in neither train nor validation or test set, since they are not included in the annotated OAC or DLFD corpus, but only occur in the Orchive. Consequently, adding such noise characteristics only to the training will most likely not improve the metrics on the test dataset. However, an improvement is expected when it comes to the evaluation of unseen Orchive tape data. The model with the best validation accuracy was picked to run on the test set. Figure 5 shows that OS2 performed slightly better than OS1. The similarities in terms of validation and test metrics between both models were expected, because those additional noise files were only added to the training set. Moreover, the validation/test data (Table 1) do not completely reflect the real situation of the Orchive. A considerable amount of very weak (silent) audio parts and special/rare noise files was observed in those tapes. Slightly better results of OS2 are primarily a consequence of the changed normalization approach. However, additional noise files had a positive effect on the analysis of the entire, enormously inhomogeneous, Orchive data. Based on the 7,447 test samples (Table 1) combined with a threshold of ≥0.5 (killer whale/noise), OS1 achieved the following results: ACC = 94.66%, TPR = 92.70%, FPR = 4.24%, PPV = 92.42%, and AUC = 0.9817. OS2 accomplished the following results: ACC = 94.97%, TPR = 93.77%, FPR = 4.36%, PPV = 92.28%, and AUC = 0.9828. For handling the extreme variety of audio signals in the ≈19,000 hours of underwater recordings, it is particularly important to have a well generalizing and robust network which can reliably segment.

### Orchive

In a next step, OS1 and OS2 were applied to all 23,511 Orchive tapes. Each tape was processed using a sliding window approach with a window size of 2 s and a step size of 0.5 s. More detailed information about all different evaluation scenarios is given in the methods section. All resulting audio segments were classified by OS1 and OS2 into “noise” or “killer whale”. The threshold for detecting “killer whale” and calculating the PPV was set to ≥0.85 for both models. Based on the detected killer whale time segments, annotation files were created in which contiguous or neighboring killer whale time segments were combined into one large segment. By having a small step size of 0.5 s and thus a high overlap of 1.5 s, neighboring segments in general were similar. To exploit this property, an additional smoothing method was introduced to deliver more robust results. Detected “noise” segments were assigned as “killer whale”, if they are exclusively surrounded by classified “killer whale” segments. Neighboring segments are segments that contain signal parts of the preceding or following overlapping time segments. This procedure removed single outliers in apparent homogeneous signal regions classified as “killer whale”. Due to the applied smoothing temporally short successive killer whale sound segments are combined into larger segments. Because of the extraordinary amount of data, manual evaluation was limited to 238 tapes (≈191.5 hours). Considering a confidence level of 95.0% with respect to 23,511 Orchive tapes corresponds to an error margin of about 6.0% when evaluating 238 tapes. For each year, a number of tapes was randomly selected, ranging from 6 to 22 per year. Every selected tape was neither included in the training nor in the validation set of OS1 and OS2. All extracted killer whale segments were manually verified by the project team. Each of the audio clips, segmented and extracted as killer whale, was listened to, and in addition visually checked by verifying the spectrograms. Time segments containing ≥1 killer whale signal were considered as TP, whereas time segments with no killer whale activation were regarded as FP. Human voice encounters were excluded from the evaluation. Table 3 visualizes the results of 238 verified Orchive tapes. In the first column (Y) each of the 23 years is displayed. The second column (T) illustrates the total numbers of processed tapes per year. The rest of the Table is separated into: detected killer whale segments (S) and metric (M). The killer whale segments were split into total, true and false killer whale segments. The extracted killer whale parts were analyzed by using two different units – samples and time in minutes. The PPV has been calculated for both models, also in a sample- and time-based way. The last row of Table 3 displays the final and overall results. The maximum clip length for OS1/OS2 was 691.0/907.5 seconds. On average, the classified killer whale segments for OS1/OS2 were about 5.93/6.46 seconds. OS1 extracted in total 19,056 audio clips (31.39 h), of which 16,646 (28.88 h) segments were true killer whale sounds and 2,410 (2.51 h) clips were wrongly classified. This led to a final sample- and time-based PPV of 87.35% and 92.00%. OS2 extracted in total 19,211 audio clips (34.47 h), of which 17,451 (32.13 h) segments were true killer whale sounds and 1,760 (2.34 h) segments were wrongly classified. This led to a final sample- and time-based PPV of 90.84% and 93.20%. As already expected, OS2 generalized better on the very heterogeneous Orchive data. Overall, with almost the same number of total detected segments, about 3.08 h (155 clips) less audio was found by OS1. A segment difference between OS1 and OS2 resulted in 805 TP and a time distinction of 3.25 h. In case of the FP, 650 different segments led to a total time disparity of 0.17 h. OS2 reduced the ≈191.5 h (238 Orchive tapes) underwater recordings to 34.47 h of killer whale events, which means roughly 18.0% of the audio data contains interesting killer whale events with an actual time of 32.13 h true killer whale sounds and 2.34 h false alarms. Extrapolating these values to the entire 18,937.5 hours of Orchive recordings, one could estimate that the entire Orchive contains roughly 3,408.75 hours of interesting killer whale signals.

**Table 3:** ORCA-SPOT segmentation results based on 238 tapes (≈191.5 hours) distributed over 23 years.

Orchive tapes																	
S & M		detected killer whale segments												metric			
		total segments				true killer whale segments				false killer whale segments				PPV (%)			
Y & T		samples		time (min.)		samples		time (min.)		samples		time (min.)		samples		time (min.)	
		OS1	OS2	OS1	OS2	OS1	OS2	OS1	OS2	OS1	OS2	OS1	OS2	OS1	OS2	OS1	OS2
1985	20	1,923	2,072	243.94	279.80	1,835	1,966	240.08	272.78	88	106	3.86	7.02	95.42	94.88	98.42	97.49
1986	7	568	492	43.44	39.40	462	478	38.54	38.84	106	14	4.90	0.56	81.34	97.16	88.72	98.58
1987	9	782	911	63.10	79.70	761	900	61.77	79.28	21	11	1.33	0.42	97.31	98.79	97.90	99.47
1988	10	690	838	66.44	90.93	631	752	63.81	84.26	59	86	2.63	6.67	91.45	89.74	96.05	92.67
1989	9	418	486	35.54	39.80	369	471	32.85	39.06	49	15	2.69	0.74	88.28	96.91	92.43	98.14
1990	10	619	585	67.41	67.18	544	577	63.08	66.89	75	8	4.33	0.29	87.88	98.63	93.57	99.57
1991	10	552	544	41.29	44.16	459	504	35.13	42.22	93	40	6.16	1.94	83.15	92.65	85.09	95.60
1992	10	680	625	58.79	58.89	591	620	54.28	58.67	89	5	4.51	0.22	86.91	99.20	92.32	99.62
1993	9	607	579	93.72	98.58	578	568	92.39	98.13	29	11	1.33	0.45	95.22	98.10	98.59	99.54
1994	9	891	899	89.50	98.13	846	870	87.79	96.83	45	29	1.71	1.30	94.95	96.77	98.09	98.68
1995	8	289	753	18.37	75.23	241	381	16.12	40.30	48	372	2.25	34.93	83.39	50.60	87.75	53.56
1996	9	516	787	48.79	62.88	374	524	30.83	42.57	142	263	17.96	20.31	72.48	66.58	63.19	67.70
1998	10	735	739	90.03	95.37	675	732	87.20	95.11	60	7	2.83	0.26	91.84	99.05	96.86	99.73
1999	10	695	763	66.86	81.47	518	548	56.91	65.53	177	215	9.95	15.94	74.53	71.82	85.12	80.43
2000	6	430	436	46.10	47.53	423	432	45.68	47.35	7	4	0.42	0.18	98.37	99.08	99.10	99.63
2001	13	1,164	1,157	109.41	117.60	1,067	1,138	102.78	116.62	97	19	6.63	0.98	91.67	98.36	93.94	99.16
2002	8	831	808	95.25	106.58	752	786	91.07	105.55	79	22	4.18	1.03	90.49	97.28	95.61	99.03
2003	10	669	710	56.94	59.88	605	697	53.68	58.98	64	13	3.26	0.90	90.43	98.17	94.26	98.50
2004	10	1,132	1,193	110.14	129.52	1,072	1,064	107.43	120.00	60	129	2.71	9.52	94.70	89.19	97.53	92.65
2005	9	1,098	1,254	106.98	147.33	975	1,032	100.98	118.87	123	222	6.00	28.46	88.80	82.30	94.39	80.68
2006	8	1,450	1,240	156.58	134.08	1,046	1,141	127.25	129.39	404	99	29.33	4.69	72.14	92.02	81.27	96.50
2009	12	1,248	1,122	106.22	104.10	955	1,060	86.68	100.63	293	62	19.54	3.47	76.52	94.49	81.60	96.67
2010	22	1,069	218	68.74	10.24	867	210	56.60	9.88	202	8	12.14	0.36	81.10	96.33	82.34	96.42
SUM	238	19,056	19,211	1,883.58	2,068.38	16,646	17,451	1,732.93	1,927.74	2,410	1,760	150.65	140.64	87.35	**90.84**	92.00	**93.20**

### ROC results orchive tapes

**Figure 6:**
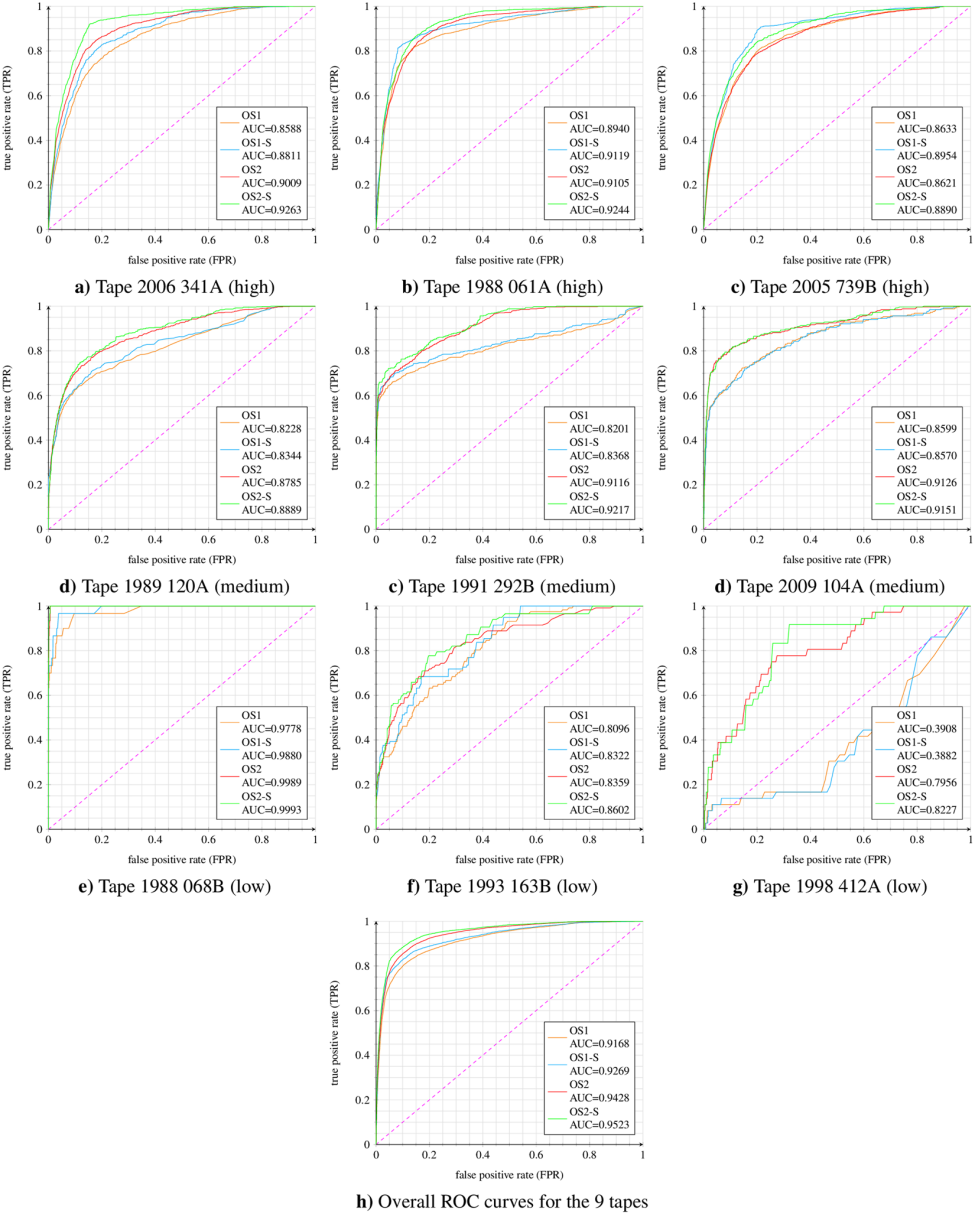
ORCA-SPOT ROC results (AUC) based on 9 (3 high, 3 mid, and 3 low killer whale activity) fully annotated Orchive tapes.

In a final step, both models were analyzed on 9 fully-annotated Orchive tapes (in total ≈7.2 h). The classification accuracy of both models, per tape and in total, was given via the AUC. The 9 tapes were chosen out of the previously selected 238 tapes based on the number of killer whale activities. Three tapes were selected with high, medium, and low number of killer whale actions. Due to our chosen sequence length of 2 seconds, combined with the selected step size of 0.5 seconds, the network classified 5,756 segments per tape. Human voice encounters were excluded from the evaluation. Human voices are spectrally similar to the killer whale pulsed calls (fundamental frequency and overlaying harmonics). Consequently the network segmented human speech as potential killer whale signals within those noise-heavy underwater recordings. Usually those sounds are not present in underwater recordings. Due to the fact that such problems are technically preventable, segmented human narrations were considered neither wrong nor correct, and were excluded from the evaluation. During manual listening of the extracted segments of the 238 tapes, all human narrations were stored in extra folders, not affecting the final result. The same was done for evaluating the fully annotated tapes. With a segment-wise comparison, all segments containing human speech were removed and discarded. The following number of killer whale events were encountered by the annotators: 2006 341A (high): 277, 1988 061A (high): 313, 2005 739B (high): 276, 1989 120A (medium): 202, 1991 292B (medium): 91, 2009 104A (medium): 77, 1988 068B (low): 6, 1993 163B (low): 11, 1998 412A (low): 14. On average, the tapes with high, medium, and low killer whale activities had 6.09, 2.60 and 0.22 annotations per minute. In addition to segment-wise comparison (OS1, OS2) a smoothed variant, based on the previously mentioned smoothing technique, was realized for both models (OS1-S, OS2-S). Figure 6 visualizes the results by presenting ROC curves and AUCs for each of the 9 tapes and also an entire ROC curve based on accumulated results of all 9 tapes. In this case, we added up the threshold-specific confusion matrices to calculate TPR and FPR. Please note that the overall ROC curve can deviate strongly from the ROC curve of the individual tapes, since the killer whale activities per tape varies by a factor of up to 95 (≈17 s versus ≈27 min per ≈45 min tape). In summary, the model OS2/OS2-S performed better, especially on noisier data considering the AUC of 0.9428/0.9523. With respect to the overall ROC curve for OS2-S (0.9523), the killer whale segmentation successfully reduced the total duration of all 9 tapes (≈7.2 h) to interesting signal parts. By extracting 93.0%, 96.0%, or 99.0% of all valid killer whale sound events the entire 7.2 hours of underwater recordings were reduced to 2.14 h, 2.91 h and 4.75 h. FPR values of 5.0%, 10.0% and 15.0% resulted in 81.9%, 88.3% and 91.9% true killer whale detections and consequently reduced the total duration to 1.30 h, 1.68 h and 2.02 h. Considering the 9 selected tapes as a representative sample of the ≈19,000.0 hours of Orchive data led to the following results: finding 96.0% of all killer whale activities reduce the Orchive to 7,653.9 h (0.87 years) whereas 5.0% false alarms and 81.9% killer whale detection shrinks the Orchive down to 3,419.3 h (0.39 years). OS2-S, based on the 9 tapes, and FPR values of 5.0%, 10.0% and 15.0% achieved accuracies of ACC = 92.82% ACC = 89.75% and ACC = 86.26%.

## Discussion

In the current study, a CNN-based pipeline was developed, in order to examine audio signals regarding certain valuable, user-specific bioacoustic events. Generalizing the pipeline makes it possible to also apply this approach to other animal species. The illustrated segmentation process is equivalent to a pre-filtering of relevant and desired acoustic events from uninteresting and superfluous signals. To improve the segmentation it is important to model the huge variety of noise. Various augmentation techniques and additional noise files were used to tackle this problem and a dB-normalization was used for OS2 in order to also handle very weak signals. Mel-spectrograms as a network input led to an excessive loss of resolution in higher frequency bands, which was a big problem considering the high-frequency pulsed calls and whistles. In addition to the selection of a suitable network architecture (Table 2), the distribution of training data is also of crucial importance. The Orchive contains much more noise than killer whale activities. It must be ensured that the training/validation dataset matches the unseen testing environment best. In order to avoid misclassifications due to an unbalanced dataset, OS2 was trained on additional noise files (5,655 very weak (silent) and 454 special/rare noises), in order to better represent the spread of noise characteristics within the Orchive. Adding those files led to a killer whale/noise ratio of 1:3 (Table 1) in the training set.

**Figure 7:**
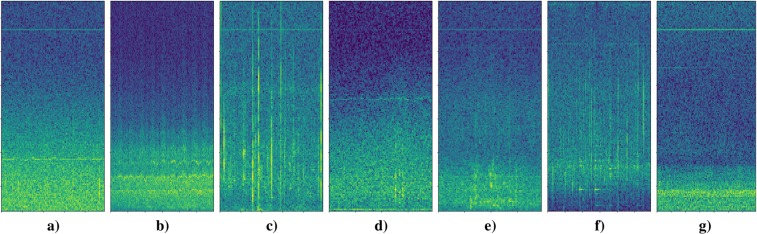
Spectrograms of noise segments classified by OS2 as potential killer whale sounds (false positives) (sampling rate = 44.1 kHz, FFT-size = 4,096 samples (≈100 ms), hop-size = 441 samples (≈10 ms), frequency range: 0–13 kHz).

During network training/evaluation several challenges were observed. One challenge is a robust detection of echolocation clicks. Echolocation clicks resemble many of the noise files and are very hard to distinguish from noise, even for human hearing (Fig. 7). The chosen FFT-size of 4,096 led to an excessive loss of accuracy in time. Smaller FFT-sizes result in large frequency resolution losses, which drastically affect the detection accuracy of pulsed calls and whistles. Another challenge is stationary boat (engine) noise. Such signals are reflected in spectrograms as stationary frequency bands. Typically, these stationary frequency bands were within the frequency ranges (1.5 kHz–18 kHz) of killer whale pulsed calls and whistles (Fig. 7). Due to the confusion between overlaying killer whale harmonics and stationary boat noises at certain frequencies such signals were often misinterpreted. However, the indicated problem did not relate exclusively to stationary boat noises. There were several encounters of other special noises (e.g. “beeps”), caused by the recording devices, which have a similar impact. Another problem observed during evaluation of the 238 tapes was a considerable amount of noise before, between, and after extracted killer whale segments. Some segments also contain overlapping vocalizations of different animals or multiple types of killer whale vocalizations.

We wanted to discuss the model results in two different ways: First, compare OS1 with OS2 according to the conducted experiments and results achieved within this work. Second, compare our best model with other bioacoustics research results. The latter, in terms of comparing the general approach and resulting metrics one-to-one with other bioacoustic studies, was not possible. To the best of our knowledge, there are no comparable published results on any known data corpus. The methodical differences between previously published individual studies which made a comparison of our results with them impossible were among others: (1) other animal species, (2) size of the dataset, (3) different methodologies, and (4) varying evaluation metrics. Therefore, our discussion of previously published studies is not a direct comparison to other work, but more or less an overview of similar studies in bioacoustics in order to show that the way of proceeding is reasonable and valid.

Figure 5 shows that training and validation metrics of both models behave similarly during training. OS1, having an AUC of 0.9817, and OS2, with an AUC of 0.9828, almost had identical results on the test set (Table 1). For both models, differing in training sample size and normalization, there are no indications of over-/underfitting (see training/validation accuracy and test set AUC in Fig. 5). Table 3 shows that OS2 outperformed OS1 on the 238 verified tapes. OS2 had fewer FP than OS1. Moreover, the detection rate of OS2 regarding the TP segments was significantly higher as well. A more robust detection of noise segments resulted in fewer misclassifications and in a more accurate detection of true noise/killer whale segments. Usually FP were single outliers surrounded by noisy signal parts. Therefore, such signal pieces normally have a much shorter duration per clip and consequently were not affected by smoothing due to isolation by adjacent noisy signal segments. Thus, a considerable difference in the number of segments only led to a very small difference in useful vocalization of killer whales time. Additionally trained noise files led to a significant reduction of such outliers. Moreover, the misclassifications regarding FN dropped as well. Detected killer whale segments were often affected by smoothing. Typically, killer whale signals are not just single events within a noisy environment. Thus, the detection of a killer whale sound, previously classified as FN, in conjunction with the smoothing technique of ORCA-SPOT, tends to result in larger segments, such as an outlying FP. Table 3 also visualizes that OS2 does not consistently perform better on all 23 years. There were outliers, such as the years 1995 and 1996, where the network performance was significantly worse. Such incidents need to be examined in order to improve network performance.

Figure 6 also demonstrated that OS2 generalized better on unseen data. The AUC deviations in Fig. 6 were considered under two different aspects: (1) AUC variations between the models (OS1 and OS2), and (2) AUC differences over the tapes. In general, the AUC deviations of OS1 and OS2 depend on the network robustness with respect to noise and consequently the ability of the model to generalize. Furthermore, the utilized dB-normalization of OS2 also had a positive impact with respect to very weak (silent) signals and potential false alarms. Both model types (OS1/OS2 and OS1-S/OS2-S) performed similar on the tapes with high killer whale activity. This was expected to some extent, since, with an increase of killer whale activity and a decrease of noise, it is primarily important to detect killer whale signals with correspondingly high accuracy rather than noise segments and vice versa. Significant differences were observed in noisier data. OS1 is trained with less noise than OS2. Consequently the killer whale/noise ratio of the training set (Table 1) of OS1 is larger and thus the model is less capable of correctly recognizing noise, resulting in more false alarms. Considering the medium tapes, OS1/OS2 delivered significantly different results. Since, in these tapes neither the killer whale nor the noise components were overrepresented, it is particularly important to consider a well-specified trade-off between killer whale/noise representations. Due to the similarities regarding the noise and killer whale distribution, such tapes reflect the actual difference between the models particularly well, as they are considered to be representative without preferring one of the two classes. A so-called representative tape depends on the desired intention (many killer whale detections versus few misclassifications). The variation in AUC over different tapes was mainly caused by (unseen) noise data, noise data superficially similar to killer whale vocalizations (e.g. high-frequent electric boat noise, different microphone settings or artefacts, noise similar to echolocation clicks, etc.) and by the total number of killer whale sounds per tape, highly affecting the impact of potential false positives (FPR) and hence the AUC. Figure 7 shows spectrograms of examples of noises superficially similar to killer whale vocalizations which were segmented as killer whale sounds. These different types of noise spectrograms reflect many of the detected false positives. The spectral envelope of those examples is undoubtedly very similar to potential killer whale sounds. Figure 7a,d are very similar to a killer whale whistle (narrow band tone without harmonics). The spectral content of 7c and 7f is very similar to the spectral content of echolocation clicks. The signal structures of Fig. 7b,e,g show some activity within the lower frequency regions that could be associated with some potential killer whale call activities. During the evaluation and detailed analysis of the false alarms, another phenomenon was discovered. Many of them had stationary frequency bands within higher frequency parts, like Fig. 7a,c,e,g. Such a signal characteristic was often confused with the superimposed high-frequency harmonics of pulsed calls or considered as whistles.

Significant differences between both models were observed especially for the tape 1998 412A. This tape contains only a few, weak, isolated, short and noisy killer whale sounds, which were really hard to identify. In addition, false positives had a very high impact on the AUC due to very few killer whale sounds in total. However, the trained noise representation and different normalization technique of OS2 generalized much better.

In summary OS2 generalizes significantly better on unseen data and is therefore much more appropriate to handle the large signal variety of ≈19,000 h underwater signals. The 9 tapes were additionally evaluated with the best ResNet50 model (Table 2). With an overall AUC of 0.9413 and 0.9519 (non-smoothed/smoothed) ResNet50 achieved almost identical results as ResNet18, which is another reason to use the much faster ResNet18 architecture.

As already mentioned, a comparison to previous research work is not so easy because there is no similar work with respect to the utilized data, methods and results. In order to emphasize the value of the work and our best network model (OS2), similar bioacoustic works were named without any direct comparison. Ness^56^ built a classifier to segment between killer whale sounds, noise and human voices. He used a dataset containing 11,041 manually labeled audio files from the Orchive tapes, sampled at 44.1 kHz. A support vector machine (SVM) using a radial basis function kernel resulted in an ACC of 92.12% using cross-validation. Grill *et al*.^86^ used CNNs for bird audio detection. The model consists of 4 convolutional/pooling-layers plus 3 fully-connected layers. It was trained on mel-scaled log-magnitude spectrograms and integrates several augmentation techniques. Grill *et al*.^86^ won the bird audio detection challenge 2018 (see Stowell *et al*.^79^) achieving an AUC of 0.9750 by using cross-validation and a final submission AUC of 0.8870 on the hidden test set. Himawan *et al*.^87^ used CNN and convolutional recurrent neural network (CRNN) detecting koala sounds in real-life environment. Both models have 3 convolutional/pooling-layers plus 2 fully-connected layers^87^. The CRNN includes an additional LSTM-layer between the convolutions and dense layers^87^. Himawan *et al*.^87^ trained on 2,181 koala and 4,337 non-koala log-scale spectrograms, sampled at 22.05 kHz. CNN (AUC = 0.9908) and CRNN (AUC = 0.9909) achieved similar results using cross-validation. Furthermore Himawan *et al*.^87^ applied both models to bird audio detection achieving AUCs of 0.8357 (CNN) and 0.8746 (CRNN). In a recent work of Google, Harvey *et al*.^57^ trained a CNN in order to detect humpback whale audio events in 15 years of underwater recordings. Harvey *et al*.^57^ used ResNet50, trained on 0.2% of the entire dataset. The model was evaluated by identifying whether a 75-second audio clip contains humpback calls. Harvey *et al*.^57^ indicated a precision over 90% together with a TPR of 90%.

This is the first study using deep learning in order to verify the general feasibility of creating a robust, reliable, machine-driven, and animal sound independent segmentation toolkit by taking the killer whale as a prototype and extensively evaluating the models on a 19,000 hour large killer whale data repository – the Orchive^55^.

During this research study, several interesting and also necessary future aspects for work have emerged. First of all, it is necessary to examine wrong classifications (see common false positives in Fig. 7) and outlying tapes in order to detect potential problems or challenges and use the cleaned data for re-training of ORCA-SPOT to ensure an iterative improvement and better generalization. Unsupervised machine-learning techniques are used to identify such common and characteristic noise misclassifications. Subsequently samples of machine-clustered noise classes are selected in order to add them to the training and/or design auxiliary preprocessing steps or slightly different model architectures to better handle such critical noise signals. In addition, it has to be considered to what extent individual calls can be extracted from the segments containing multiple calls, how to remove the remaining noise in the segments, and how to deal with overlapping calls. Consequently, fine tuning of the already existing segments is a very important aspect. In order to further reduce remaining/surrounding noise within pre-segmented killer whale segments or to split up segments containing multiple killer whale sounds into single-sound segments, an iterative segmentation approach (shorter sequence length and step size) is a possible solution to create finer structures. Nevertheless, overlapping calls will still be included in one segment. It is important to first identify and encapsulate all these segments in a post-process, e.g. via unsupervised clustering, in order to avoid any negative impact of such segments regarding potential call type classification training. A call type classifier trained on machine-identified and clustered killer whale call types, by using the large amount of pre-segmented signals, is a possible method to identify potential call types in such overlapping structures in order to separate them somehow. While this study focuses on a pure segmentation between potential killer whale sounds and various noises (binary classification problem), first and prelimnary experiments/results on call type classification have already been carried out^94^. A ResNet18-based classifier was trained on a small dataset in order to classify 12 different classes of vocalizations (9 call types, whistles, echolocation clicks, and noise). The resulting call type classifier achieved a mean test set accuracy of 87.0% on a 12-class problem^94^. In addition, the extracted segments from 19,000 hours of underwater recordings provide a very powerful basis for various automatic, fully unsupervised machine-learning approaches, e.g. representation learning followed by clustering to derive machine-identified killer whale call types. At the same time, many other advantages would also arise here: (1) no data annotation required, (2) eliminating human errors (e.g. labeling based on human perception, misclassifications, etc.), (3) analysis of large data corpora possible, and (4) deriving potential unknown killer whale call type structures, e.g. sub-call types.

In future work, we will also have to evaluate whether it is better to train the echolocations in a separate network. In addition, the scope of future research will be broadened to include experiments on different and optimized network architectures. There should be also investigations in the field of CRNN in order to tackle problems of how to differentiate between stationary and varying frequency characteristics (e.g. caused by electric boat noise). Both problems become particularly clear in Fig. 7. Furthermore, it is useful to investigate ResNet50 and its detection accuracy. Further detailed call analyses, combined with the collected video recordings and behavioral descriptions, accumulated in the project DeepAL by various biologists, offer possibilities to gain a deeper understanding of killer whale communication. Considering all the above-mentioned future work, the maintenance of the current pipeline needs to be ensured, in order to present a stand-alone system, which can be adapted to a variety of bioacoustical data corpora, together with the corresponding training data. Last but by no means least, ORCA-SPOT will subsequently be prepared to be deployed in July 2019 in British Columbia as a quasi real-time killer whale detection system during the fieldwork. Further evaluation regarding the extent to which ORCA-SPOT can be able to assist the search of the animals efficiently and purposefully will be conducted on the field mission in July 2019.

To summarize, ORCA-SPOT allows a robust pre-segmentation of large bioacoustic datasets into relevant and irrelevant signal parts. Researchers can concentrate on those sub-data containing only interesting bioacoustic events. According to the OS2-S overall ROC curve and the results based on the 238 evaluated 45-minute tapes, 80% of all killer whale activations and 5% misclassifications reduced the whole Orchive by about 80% to 0.4 years.

## Methods

This section describes network architectures, methods, and algorithms used for training and implementation of ORCA-SPOT. Besides a brief overview about the ORCA-SPOT architecture, data preprocessing, network training, network evaluation and testing is explained.

### Convolutional neural network (CNN)

Convolutional Neural Network (CNN) is an end-to-end deep neural network architecture in machine learning that is able to efficiently handle the complexity of 2-dimensional input data (e.g. spectrograms)^95^. CNNs are built on the principle of pattern recognition and consist of a feature extraction/learning component and a classification part^95,96^. The convolutional layers are responsible for feature learning/extraction and are characterized by three significant architectural concepts: local receptive fields, shared weights and spatial or temporal subsampling (pooling)^95^. Convolving the kernel over the entire input by a defined shifting size (stride), covering a certain receptive field, results in multiple (hidden) units, all sharing the same weights and combined together in one single feature map^95^. Usually a convolutional layer consists of multiple feature maps (channels) in order to learn multiple features for the same position^95^. CNN architectures include pooling layers to reduce the resolution of a feature map by calculating a localized statistic. Convolutional layers only calculate linear operations. Thus, a non-linear layer using an activation function, usually the Rectified Linear Unit (ReLU)^97^ function, is added. Furthermore, a normalization layer (e.g. batch normalization^98^) is added to ensure a stabilized distribution of the activation values^98^. The extracted and learned features based on several, repetitive and configurable sequences of convolutional, normalization, activation, and pooling layer, are now projected onto the corresponding output classes using one or more fully connected layers. Consequently, the fully connected layers are responsible for the classification.

#### ORCA-SPOT architecture

A network consisting of concatenated residual layers (see He *et al*.^93^) is called residual network (ResNet). In practice there exist different and approved ResNet architectures (see He *et al*.^93^), based on the number of concatenated layers. A detailed description about deep residual learning in general can be found in the work of He *et al*.^93^. Figure 4 visualizes the proposed ORCA-SPOT architecture corresponding to the established ResNet18^93^ architecture, except that in the first residual layer the max-pooling layer was removed. The main intention was to process the data with a preferably high resolution as long as possible. This max-pooling layer in combination with a stride of 2 leads to a big loss of resolution already at the initial stage. This is a disadvantage regarding high-frequency subtle killer whale signals. After the last residual layer, global average pooling is performed on the bottleneck training features, consisting of 512 feature maps with 16 × 8 hidden units. These results are now connected to a 512-D fully connected layer, projecting its output onto two output classes: “killer whale” and “noise”.

### Data preprocessing and training

ORCA-SPOT converts every audio clip into a 44.1 kHz mono wav-signal. The remaining signal was transformed to a power spectrogram using a fast Fourier transform (FFT) using a FFT-size of 4,096 samples (≈100 ms) and a hop-size of 441 samples (≈10 ms). In a next step the power spectrogram was converted to decibel (dB) scale. Based on the chosen sampling rate and FFT-size each training file was represented by a 2,049 × T feature matrix, where T represents the time dimensionality of the input. In order to obtain the largest possible variety of training variants and to simultaneously handle available disk space, the augmentation was performed in an embedded way rather than generating augmented samples on the hard disk. The augmentation used the previously decibel-converted power spectrogram as input. All augmentation techniques were processed on-the-fly. The augmentation was computationally very expensive because of various random sampling/scaling operations. Consequently, this was implemented using PyTorch^99^ multiprocessing in order to process the entire pre-processing on the CPU in parallel, whereas the network training utilized the GPU. In a first step intensity, pitch, and time augmentation were conducted. Random scalings based on a uniform distribution were performed within predefined ranges: amplitudes/intensity (−6 dB–+3 dB), pitch factor (0.5–1.5), and time factor (0.5–2.0). The frequency dimensionality of the augmented spectral result was compressed by using a linear frequency compression (nearest neighbor, fmin = 500 Hz, fmax = 10 kHz). The number of frequency bins was reduced to 256, resulting in a final spectral shape of 256 × T. In a second augmentation step noise augmentation was carried out. A pitch- and time-augmented frequency-compressed noise spectrogram from the training set was added to the spectrogram using a random-scaled (uniformly distributed) signal-to-noise ratio (SNR) between −3 and +12 dB. Longer noise files were cut and shorter noise signals were self-concatenated in order to match the time dimensionality of the training spectrogram. The noise augmentation is followed by a dB-normalization (min = −100 dB, ref = +20 dB) between 0 and 1. For a successful training process, it is essential to have equally-sized (frequency and time dimensionality) training data. Consequently, the current spectral shape of 256 × T requires a constant time domain. This was solved by randomly subsampling or padding the resulting training spectrogram (256 × T) being longer or shorter than 1.28 s in order to derive a final trainable spectral shape of 256 × 128.

In summary, the following data preprocessing/augmentation pipeline, implemented in PyTorch^99^, was realized by ORCA-SPOT: convert audio to mono, resampling to 44.1 kHz, power spectrum, dB-conversion, intensity augmentation, pitch augmentation, time augmentation, linear frequency compression, noise augmentation, dB-normalization, and accidental subsampling/padding to get a trainable clip for the ORCA-SPOT network. In order to be able to compare the validation/test set to multiple models, shorter/longer validation and test signals than 1.28 s were always centered and not randomly extracted/padded. The model was trained and implemented using PyTorch^99^. ORCA-SPOT uses an Adam optimizer with an initial learning rate of 10^−5^, *β*_1_ = 0.5, *β*_2_ = 0.999 and a batch-size of 32. After four epochs and no improvements concerning the validation set, the learning rate decayed by a factor of 1/2. The training stopped if the validation accuracy did not improve within 10 epochs. Finally, the model with the best validation accuracy was selected. The test set was only used to evaluate the final model performance and was neither involved in the training nor in the validation.

### Evaluation and testing

ORCA-SPOT was verified on two different test scenarios. On the one hand, the model was evaluated on the test data described in Table 1, and on the other hand ORCA-SPOT was applied to the 23,511 Orchive tapes (≈18,937.5 hours). In the first case there were already labeled test audio clips as a benchmark, provided as input to the model using a centered 1.28 s window. In the second case, the raw Orchive tapes were evaluated. Audio clips of a given configurable sequence length (2 s) and step size (0.5 s) were extracted and fed in its entirety (without centering) to the network. Each of the audio clips resulted in a 1 × 2 probability matrix that the given signal segment was a killer whale or noise. Consecutive killer whale/noise predictions were concatenated to one audio slice of multiple calls or noise segments. It is of great importance that the network is able to process the ≈2.2 years of audio in finite time. The prediction time of the network was adapted and optimized in combination with a mid-range GPU (Nvidia GTX 1050). For calculating the area-under-the-curve (AUC) and other metrics (accuracy (ACC), true-positive-rate (TPR), false-positive-rate (FPR), positive-predictive-value (PPV)) we used Scikit-learn^100^, an open-source machine-learning library in Python.

## Data Availability

The Orchive data and the Orchive annotation catalog (OAC) used in this study are available upon request only in agreement with the OrcaLab^55^ and Steven Ness^56^. Following the open science principles, the source code and the DeepAL fieldwork data 2017/2018 (DLFD) are planned to be made freely available^88,90^ to the research community and citizen scientists in October 2019 after the current pilot study concludes. Furthermore, all segmented and extracted audio samples, which result from this study, will be handed over to the OrcaLab^55^ and Steven Ness^56^.
